# Effectiveness of using e-government platform “Absher” as a tool for noncommunicable diseases survey in Saudi Arabia 2019–2020: A cross-sectional study

**DOI:** 10.3389/fpubh.2022.875941

**Published:** 2022-09-21

**Authors:** Mohammed Alluhidan, Reem F. Alsukait, Taghred Alghaith, Rana Saber, Adwa Alamri, Saleh Al-Muhsen, Fahad Alhowaitan, Abdulmohsen Alqarni, Christopher H. Herbst, Nahar Alazemi, Ahmad S. Hersi

**Affiliations:** ^1^General Directorate for National Health Economics and Policy, Saudi Health Council, Riyadh, Saudi Arabia; ^2^Division of Health Research, Lancaster University, Lancaster, United Kingdom; ^3^Community Health Department, King Saud University, Riyadh, Saudi Arabia; ^4^Department of Pediatrics, King Saud University, Riyadh, Saudi Arabia; ^5^Department of Business Analysis, Elm, Saudi Arabia; ^6^Health, Nutrition, and Population Global Practice Group, World Bank, Washington, DC, United States; ^7^Cardiac Science Department, King Saud University, Riyadh, Saudi Arabia

**Keywords:** NCDS, e-health, cross-sectional, Saudi Arabia, e-government platforms, surveillance

## Abstract

**Background:**

E-government platforms provide an opportunity to use a novel data source for population health surveillance (also known as e-health). Absher is a Saudi e-government platform with 23 million authenticated users, including residents and citizens in Saudi Arabia. All Absher users were invited to participate in a web-based survey to estimate the prevalence of noncommunicable diseases and their risk factors in Saudi Arabia.

**Objective:**

To assess the potential of using an e-government platform (Absher) to administer web-based health surveys.

**Methods:**

A cross-sectional, web-based health survey was administered to Absher users between April 2019 and March 2020. The survey instrument included eight items and took <5 min to complete. The respondents' data were compared to Saudi Arabia's 2016 census. Descriptive summary statistics of the prevalence of major noncommuncable diseases are presented and compared to population-based prevalence data from Saudi Arabia's World Health Survey (WHS) 2019. All analysis was conducted using Stata 13.0.

**Results:**

Overall, the Absher health survey had a 24.6% response rate, with most respondents being male (84%), Saudi (67%), and between 30 and 44 years of age (49%). Overall, the prevalence of noncommunicable diseases and risk factors among respondents was high for overweight (35%) and obesity (30%) and low for asthma (6%). The prevalence of diabetes, dyslipidemia, and hypertension was between 15 and 17% on average, and 26.5% were smokers. In comparison to population-based World Health Survey estimates, the Absher survey overestimated obesity, diabetes, dyslipidemia, hypertension, and smoking rates, and underestimated overweight, whereas asthma prevalence was similar for Absher and the WHS.

**Conclusions:**

With improvements in the study design, the use of e-government platforms can provide a useful and potentially low-cost data source for public health research.

## Introduction

Public health surveillance offers a systematic approach for the regular collection and routine analysis of health indicators over time, and comparisons between different population groups. It allows for the regular dissemination of results and sharing of available scientific knowledge (1). One of the key aims of public health surveillance is to allow policymakers to determine the burden of disease in a timely manner to formulate policies and programs that mitigate the impact of diseases and their risk factors. Another key aim is to measure the progress and efficacy of preventive efforts already implemented (1, 2). However, traditional public health surveillance systems can be costly, and can also involve significant delays between data collection and dissemination of results (3).

In 2018, the World Health Assembly acknowledged the potential of digital technologies to play a major role in improving public health. *E-government* can be defined as the use of information and communication technology (ICT) to more effectively and efficiently deliver government services to citizens and businesses (4). There has been global interest in the potential of ICT for health. The term *e-health* encompasses the cost-effective and secure use of ICT in support of health and health-related fields, including healthcare services, health surveillance, health literature, and health education, knowledge, and research (5). As part of the 2020 Riyadh Declaration on Digital Health, innovative surveillance systems were highlighted as a core component of the connected global health system (6).

In 2003, Saudi Arabia started its digital transformation to e-governance and began offering government services electronically (7). Absher provides the essential infrastructure that underpins Saudi Arabia's e-government services. Since its launch in 2010, Absher has developed into a three-dimensional framework for individuals, businesses and government entities (8). Absher Individuals has enabled citizens and residents of Saudi Arabia to access a broad collection of e-services provided by different government agencies (for example, the Ministry of Civil Affairs, Ministry of Housing, and General Directorate of Traffic). Absher registration requires a national or residence ID number, fingerprint biometrics authentication and a valid mobile number. The user account is then activated through Absher registration by visiting an activation office (passports or civil affairs offices), a bank, or a self-service machine (8). The 23 million registered users of Absher can access more than 330 different e-government services including driving license renewal, disputing traffic violations, issuing and renewing the Saudi passport, issuing and renewing visas and residence permits, reporting missing documents, authentication of lease contracts and newborns registration (8).

Noncommunicable diseases (NCDs) are a major health and economic burden in Saudi Arabia (9). They are currently the leading cause of death and disability-adjusted life years in the country, accounting for 66.7% of deaths (10). The NCD burden will likely continue to rise with the country's demographic transitions since NCDs disproportionately affect older people. Vision 2030 is Saudi Arabia's strategic framework to reduce the country's dependence on oil, diversify its economy, and develop public service sectors, such as health, education, infrastructure, recreation, and tourism (11). Through the Health Sector Transformation Plan, the vision outlines a number of strategic objectives to improve life expectancy and realize economic potential in the country, including the promotion of health prevention.

However, there are gaps in the data, considering that the last two population-based national surveys were the 2013 Saudi Health Interview Survey (SHIS) and the Saudi Arabia's World Health Survey (WHS) in 2019 (12, 13). Given the significant time lag between these surveys, there is a need for more frequent data collection to prioritize policy actions and allocate resources (14). Web-based surveys are typically quicker to complete and cost less per participant than other survey modes (15). Public health professionals are highly encouraged to develop and experiment with new digital innovation tools for health-related data collection (16). Absher platform can provide such a digital tool.

Between 2019 and 2020, a web-based survey was launched using Absher's e-government platform as a novel way to collect health-related data and determine the burden of NCDs in Saudi Arabia. The objectives of this paper are to (1) describe the results of the Absher survey and (2) compare the prevalence estimates collected from the Absher survey with those of a national population-based survey. The overall objective is to inform the future use of e-government tools for public health research. This work is related to the first author's master's thesis (17).

## Methods

A web-based cross-sectional health survey was launched through the Absher e-government platform (hereafter referred to as Absher) between April 2019 and March 2020. All Absher users who logged into the platform during the study period-whether they used a website or a mobile application-received an invitation (Appendix B in Supplementary material) to participate in the survey 3,584,422. Only those Absher users who consented to participate were redirected to the online survey. Ethical approval for the Absher survey was obtained from the King Fahad Medical City Institutional Review Board.

The prevalence estimate results from the Absher survey were then compared to the results from Saudi Arabia's 2019 WHS report (13). The WHS is a nationally representative population-based, cross-sectional household- and individual-level survey of adults aged 15 and older. It includes anthropometric measurements (weight, height, hip, and waist circumference), a blood pressure test, and a blood test including random plasma glucose, cholesterol, high-density lipoprotein (HDL), low-density lipoprotein (LDL), and hemoglobin for individual-level respondents (*n* = 8,912). Out of the 10,000 selected households, a total of 9,652 were present (or occupied), and 9,339 were successfully interviewed, yielding a response rate of 96.8%. In the households interviewed, one person aged 15 years or older per household was identified for individual interviews. Interviews were completed with 8,912 individuals, yielding a response rate of 95.4%. WHS IRB approval was obtained from the General Directorate for Research and Studies at the MoH (13). Additional details on the methodology and study instruments are described elsewhere (13).

## Measures

The Absher web-based survey consisted of nine questions that were available in both English and Arabic. The first five questions were yes/no questions that asked respondents whether they had been diagnosed with diabetes, hypertension, high blood cholesterol, or asthma, and whether they smoked. The next two questions were dropdown questions that asked participants to indicate their weight in kilograms and their height in centimeters. The final two questions also used a dropdown box to ask for region and city of residence. All questions are included in Appendix A in Supplementary material. Age, gender, and nationality were automatically verified through the Absher database. Only adults over the age of 15 were included in the analysis (*n* = 8,82,746). No other information was obtained, and the data were deidentified.

Absher survey data cleaning involved reorganizing existing variables, such as nationality (Saudi or non-Saudi), region (central, western, eastern, southern, and northern), and age into specific age groups (15–29, 30–44, 45–59, 60+). Additional new variables were created, including *body mass index (BMI; underweight* if BMI is <18.5; *normal* if BMI is between 18.5 and 24.9; *overweight* if BMI is between 25.0 and 29.9; or *obese* if BMI is ≥30) (18). Biologically implausible measurements (height < 111.8 cm [<44 inches] or >228.6 cm [>90 inches] and weight <24.9 kg [<55 pounds] or >453.6 kg [>1,000 pounds]) were excluded (18).

## Statistical analysis

Saudi Arabia's 2016 population census demographics (gender, nationality, age groups, and region) were used to adjust the weights of Absher survey responses. Specifically, we used raking methodology in Stata 13.1 (19). In which the weighting variables were raked according to their marginal distribution. Respondents with missing data were excluded from the analysis. Data analysis included creating descriptive summaries for each disease or risk factor by age, gender, nationality, and region of residence using Stata 13.1. Prevalence estimates from the Absher survey were then compared to the 2019 WHS report.

## Results

Overall, the majority of Absher survey respondents were male (83.7%), Saudi nationals (66.8%), between 30 and 44 years of age (49.4%), and from the more populated central and western regions (32.6 and 31.1%, respectively; Table 1).

**Table 1:** Demographic characteristics of Absher survey respondents 2019–2020 compared to Saudi Arabia's 2016 census.

	**Demographics**	**Percentage (%)**
	Absher survey	2016 Census
*N*	707,703	31,742,308^b^
**Gender**		
Male (*n*)	83.7% (592,663)	57.4% (18,233,964)
Female (*n*)	16.3% (115,040)	42.6% (13,508,344)
**Nationality**		
Saudi (*n*)	66.8% (472,610)	63.2% (20,064,970)
Non-Saudi (*n*)	33.2% (235,093)	36.8% (11,677,338)
Age group		
15–29 years (*n*)	26.6% (188,195)	24.3% (7,713,380)
30–44 years (*n*)	49.4% (349,837)	29.9% (9,205,269)
45–59 years (*n*)	18.9% (133,791)	15.5% (476,1346)
60+ years (*n*)	5.1% (35,880)	5.5% (1,745,826)
**Regions^a^**		
Central region (*n*)	32.6% (230,915)	29.6% (9,395,723)
Western region (*n*)	31.1% (220,435)	32.8% (10,411,477)
Eastern region (*n*)	19.4% (137,111)	15.1% (4,761,346)
Southern region (*n*)	11.1% (78,227)	14.9% (4,729,603)
Northern region (*n*)	5.8% (41,015)	7.7% (2,444,157)

a Central includes Riyadh and Qassim; Western includes Makkah and Madina; Eastern includes Eastern Provinces; Southern includes Asir, Jazan, Najran, and Bahah; Northern includes Tabuk, Hail, Jawf, and Northern Borders.

b Includes those under 15 years of age.

The overall prevalence of NCDs and risk factors among survey respondents was relatively high for overweight and obesity (35 and 29.9%, respectively) and lowest for asthma (6.1%). The prevalence of diabetes, dyslipidemia, and hypertension was, on average, between 15 and 17%. Based on demographic variables, regional variations in the prevalence of most NCDs and their risk factors were small (1–3% differences), except for smoking prevalence in the southern regions, which was the lowest at around 20.7% compared to 31.4% in Northern region. Both overweight and obesity prevalence rates were lower among females than males (25.9% compared to 30.7%, respectively, for obesity). Overweight was higher among non-Saudis than Saudis (42.2% compared to 31.9%, respectively). However, for non-Saudis, the prevalence of diabetes, dyslipidemia, and hypertension was approximately 7% lower than Saudis at around 11%, compared to 17–19% for Saudis. The prevalence of smoking was much higher among males than females (30.6% compared to 5.3%, respectively). Similarly, smoking was less prevalent among non-Saudis than Saudis (19.5% compared to 30%; Table 2). Additional results disaggregated by age and gender are provided in Appendix C in Supplementary material.

**Table 2:** Prevalence of noncommuncable diseases and their risk factors among Absher survey respondents 2019–2020.

**Demographic variables**	***N***	**Overweight^a^ % (number)**	**Obesity^a^ % (number)**	**Diabetes % (number)**	**Dyslipidemia % (number)**	**Hypertension % (number)**	**Asthma % (number)**	**Smoking % (number)**
Male	592,663	36.6% (216,708)	30.7% (182,032)	15.4% (91,506)	17.5% (103,906)	16.3% (96,865)	5.8% (34,533)	30.6% (181,633)
Female	115,040	29% (33,372)	25.9% (29,739)	11.7% (13,504)	14.1% (16,262)	13.2% (15,217)	7.6% (8,711)	5.3% (6,059)
**Nationality**								
Saudi	472,610	31.9% (150,856)	30.2% (142,610)	16.8% (79,628)	19.3% (91,268)	17.8% (84,238)	7.7% (36,181)	30% (141,751)
Non-Saudi	235,093	42.2% (99,224)	29.4% (69,161)	10.8% (25,382)	12.3% (28,900)	11.8% (27,844)	3% (7,063)	19.5% (45,941)
**Age groups**								
15–29 years	188,195	26% (49,002)	19.8% (37,177)	7.2% (13,639)	6.7% (12,588)	8.5% (15,980)	7.5% (14,038)	26.8% (50,400)
30–44 years	349,837	38.1% (133,179)	31.5% (110,055)	11.4% (39,735)	14.5% (50,812)	12.6% (43,994)	5.6% (19,625)	28.8% (100,847)
45–59 years	133,791	40.3% (53,941)	38.3% (51,287)	26.2% (35,090)	30.4% (40,693)	26.2% (35,097)	5.4% (7,230)	23.1% (30,943)
60+ years	35,880	38.9% (13,958)	36.9% (13,252)	46.1% (16,546)	44.8% (16,075)	47.4% (17,011)	6.6% (2,351)	15.3% (5,502)
**Region^b^**								
Central region	230,915	36.7% (84,829)	29.8% (68,871)	13.8% (31,794)	17.4% (40,093)	14.7% (33,969)	7.2% (16,554)	25.8% (59,530)
Western region	220,435	34.4% (75,776)	30.7% (67,565)	15.9% (35,061)	17.5% (38,605)	16.6% (36,586)	5.8% (12,719)	28% (61691)
Eastern region	137,111	37% (50,721)	30.9% (42,430)	15.2% (20,880)	17.6% (24,071)	17.1% (23,404)	4.8% (6,638)	27.3% (37,407)
Southern region	78,227	32.1% (25113)	27.2% (21307)	14.3% (11169)	14.2% (11,089)	15.2% (11,873)	6.3% (4,923)	20.7% (16,188)
Northern region	41,015	33.3% (13,641)	28.3% (11,598)	14.9% (6,106)	15.4% (6,310)	15.2% (6250)	5.9% (2,410)	31.4% (12,876)
Overall	707,703	35.3% (250,080)	29.9% (211,771)	14.8% (105,010)	17% (120,168)	15.8% (112,082)	6.1% (43,244)	26.5% (187,692)

a Overweight and obesity are restricted to those above 18 years of age.

b Central includes Riyadh and Qassim; Western includes Makkah and Madina; Eastern includes Eastern Provinces; Southern includes Asir, Jazan, Najran, and Bahah; Northern includes Tabuk, Hail, Jawf, and Northern Borders.

**Figure 1:**
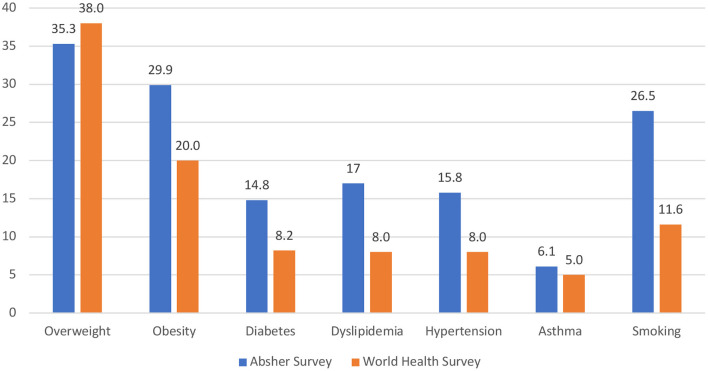
Difference in prevalence rates (%) of selected self-reported diseases and risk factors between the Absher Survey 2019–2020 and World Health Survey 2019.

When comparing Absher survey results with the 2019 population-based WHS for Saudi Arabia results, major differences appeared, as shown in Figure 1. Most notably, the Absher survey overestimated obesity, diabetes, dyslipidemia, hypertension and smoking rates by 7–15%. Asthma prevalence estimates were close in both, and overweight was underestimated in the Absher survey (35.3% compared to 38% in the WHS; Figure 1).

## Discussion

Overall, the Absher survey response rate was 24.6%, which indicates a 75% nonresponse bias. Information on non-respondents or people who logged into Absher during the survey period and did not opt in is unknown. However, the response rate was calculated based on the number of active Absher users during that period, which might overestimate the nonresponse rate, considering that unless a user needed a specific government service during that period, they likely did not log in to see the survey invitation. Another factor to contemplate is the influence of people's perceptions of public health surveillance because this survey was the first attempt in Saudi Arabia. Response rates are typically calculated by dividing the number of responses by the total number of eligible respondents in the sample (20). While mixed-mode approaches could yield higher response rates (60% compared to 51% for web-based only), paper mail is not typically used in Saudi Arabia (15).

While higher response rates indicate a higher quality survey research study, representativeness is also a major component (21). In terms of the representativeness of Absher survey respondents, there are some variations between the 2016 Saudi census and the Absher survey. Nationality status (Saudi vs. non-Saudi), age group, and region of residence were similar to the census population distribution (22). However, females were significantly underrepresented; only 16.3% of survey respondents were female, compared to the national overall estimate of 43% (22). In addition, those between 30 and 44 years of age were overrepresented among the Absher survey respondents at 49.4%, compared to 30% in the national census (22). This lack of representation could be explained by a number of factors. Until 2018, women were not allowed to have individual accounts on the Absher system. This has changed, restrictions have been removed, and women can now create their own individual Absher accounts. The rate of female users has been consistently increasing since then (23). Additionally, the number of authenticated accounts on Absher has been increasing steadily, from 7 million at the time of the survey to over 23 million at the time of writing (24). This will likely improve the representativeness of the sample in future studies.

There are differences in the prevalence rates found in the Absher survey and in the population-based WHS results. WHS diabetes and prediabetes prevalence estimates based on randomly measured blood glucose levels are close to the Absher survey results (15% compared to 14.8%, respectively; (13). Similarly, WHS hypertension based on measured blood pressure is similar to the Absher results [15.8% compared to 15.7%, respectively; (13)]. WHS dyslipidemia based on raised serum cholesterol is much higher at 43% than the 17% self-reported among Absher survey respondents (13). One potential explanation is that self-reported responses typically underestimate the prevalence of disease, as observed in the WHS's own self-reported vs. measured responses (13). Specifically, a 2013 population-based survey in Saudi Arabia found that 65% of Saudis with hypercholesterolemia and 58% of hypertensive and diabetic Saudis were undiagnosed, and therefore unlikely to be captured by self-reported data (25–27). Adding a health interview component to those who opt in to take the survey could improve the validity of the findings by allowing objective measurements of blood and anthropometrics, thereby reducing the reliance on self-report (28). Nonetheless, the use of web-based e-government platforms, such as Absher, could be an effective tool for answering subjective health-related research questions that are primarily based on self-report.

The implementation of internet-based public health surveillance systems is an emerging development in healthcare (29). Until now, most relevant studies have focused on public health surveillance systems to mitigate the impacts of infectious diseases in developing settings, establishing a gap in literature that focuses on NCDs in high-income nations (30, 31). Consequently, the existing research on NCDs surveillance, which is deployed to provide early warning detection, detect patterns in the generated information, and produce sufficient data to inform evidence-based practice, does not cover all critical areas in high-income countries (30).

## Strengths and limitations

The Absher survey has several strengths, including having authenticated users with age, region, and nationality data generated by the system from the e-government platform using national ID numbers. Additionally, the survey was web-based, so it was neither cost- nor labor-intensive. The data required little cleaning (32). Future studies could estimate the cost per respondent using Absher compared to other outlets, such as web panels. The survey itself also had a low time commitment for respondents, as it took <5 min to complete. Studies have shown that time commitment is an important factor in improving response rate (33). Finally, to address the skewness of the data's lack of representativeness, the findings were weighted based on demographics from the national census. However, there were little differences between adjusted and unadjusted results.

Some of the limitations of using Absher as a tool for web-based surveys have been touched on in the discussion. Mainly, it had a low response rate, which could have resulted in nonresponse bias. It was difficult to obtain information on non-respondents because of ethics and privacy considerations. Future studies could ensure that information on the number of people who logged into Absher and saw the survey is captured to enable comparisons between responders and nonresponders and assess whether there are significant differences between the two groups. One potential bias is whether there are any disparities in accessing the internet thereby the platform. Nonetheless, recent national surveys show that ICT in KSA is high with 96.8% of households having internet access and 99.1% have mobile phone access in 2020 (34). Additionally, the low female participation rate should be addressed in future surveys. The survey questions were not validated, and future studies could improve their strength by using validated tools. For example, improvement could include conducting health interviews with blood samples from a subset of users to improve the validity of results in comparison to self-reported data. Finally, optimizing the use of Absher by linking the survey responses to other data available in the Ministry of Health's Sehaty database using the national or resident permit ID numbers could enable many research questions to be answered.

## Conclusion

Digital health applications, such as those linked to e-government services, can serve as valuable data collection tools. Absher offers a unique platform for ongoing cross-sectional studies. Its possibilities and applications are far-reaching and continue to evolve as a potential asset to public health.

## Data availability statement

The raw data supporting the conclusions of this article will be made available by the authors, without undue reservation.

## Ethics statement

The studies involving human participants were reviewed and approved by King Fahad Medical City IRB Log Number: 20-425E. Written informed consent from the participants' legal guardian/next of kin was not required to participate in this study in accordance with the national legislation and the institutional requirements.

## Author contributions

MA, TA, AH, and NA conceived the idea. MA, RA, FA, and AAlq procured the relevant datasets. RA led the data analysis. MA, RA, and CH took the lead in organizing, writing, and revising the manuscript. All authors contributed, read, and approved the final manuscript.

## Conflict of interest

Authors FA and AAlq were employed by Elm.

The remaining authors declare that the research was conducted in the absence of any commercial or financial relationships that could be construed as a potential conflict of interest.

## Publisher's note

All claims expressed in this article are solely those of the authors and do not necessarily represent those of their affiliated organizations, or those of the publisher, the editors and the reviewers. Any product that may be evaluated in this article, or claim that may be made by its manufacturer, is not guaranteed or endorsed by the publisher.

## Abbreviations

ICT: information and communication technology
NCD: noncommunicable disease
WHS: World Health Survey.
