# Supplementary material for: Effectiveness of using e-government platform “Absher” as a tool for noncommunicable diseases survey in Saudi Arabia 2019–2020: A cross-sectional study
Source: Front Public Health. 2022 Sep 21;10:875941. doi: 10.3389/fpubh.2022.875941 (PMC9534281; doi:10.3389/fpubh.2022.875941)
Supplement: Supplementary file 1 [file Data_Sheet_2.PDF]

Chronic diseases survey

The Saudi Health Council is conducting a national survey in Absher platform to determine the burden of disease in The Kingdom. The survey consists of simple questions that shouldn't take you more than one minute to complete.
Would you want to participate in this survey?

- (●) Yes
- ( ) Participate later
- ( ) I do not want to participate

Next

STC 10:40 pm 35%
absher.sa

VISION 2030 BETA

Chronic diseases survey

Dear respondents,

Your participation will help us to improve the provision of healthcare services and form part of the national decision-making process.

Yours truly,
Tawfiq Alrabiah
Minister of Health/ Chairman of the Saudi Health Council

1 **Have you been diagnosed with diabetes?**
- Yes
- No

2 **Have you been diagnosed with hypertension?**
- Yes
- No

3 **Have you been diagnosed with high blood cholesterol?**
- Yes
- No

4 **Do you smoke?**
- Yes
- No

5 **Do you have asthma?**
- Yes

STC 10:40 pm 35%

absher.sa

BETA

3 Have you been diagnosed with high blood cholesterol?

- Yes
- No

4 Do you smoke?

- Yes
- No

5 Do you have asthma?

- Yes
- No

6 Weight (kg)

Please Select

7 Height (cm)

Please Select

8 Region

Please Select

9 City of Residence

Please Select

Submit
