# Supplementary figures and images for: Effectiveness of using e-government platform “Absher” as a tool for noncommunicable diseases survey in Saudi Arabia 2019–2020: A cross-sectional study
Source: Front Public Health. 2022 Sep 21;10:875941. doi: 10.3389/fpubh.2022.875941 (PMC9534281; doi:10.3389/fpubh.2022.875941)

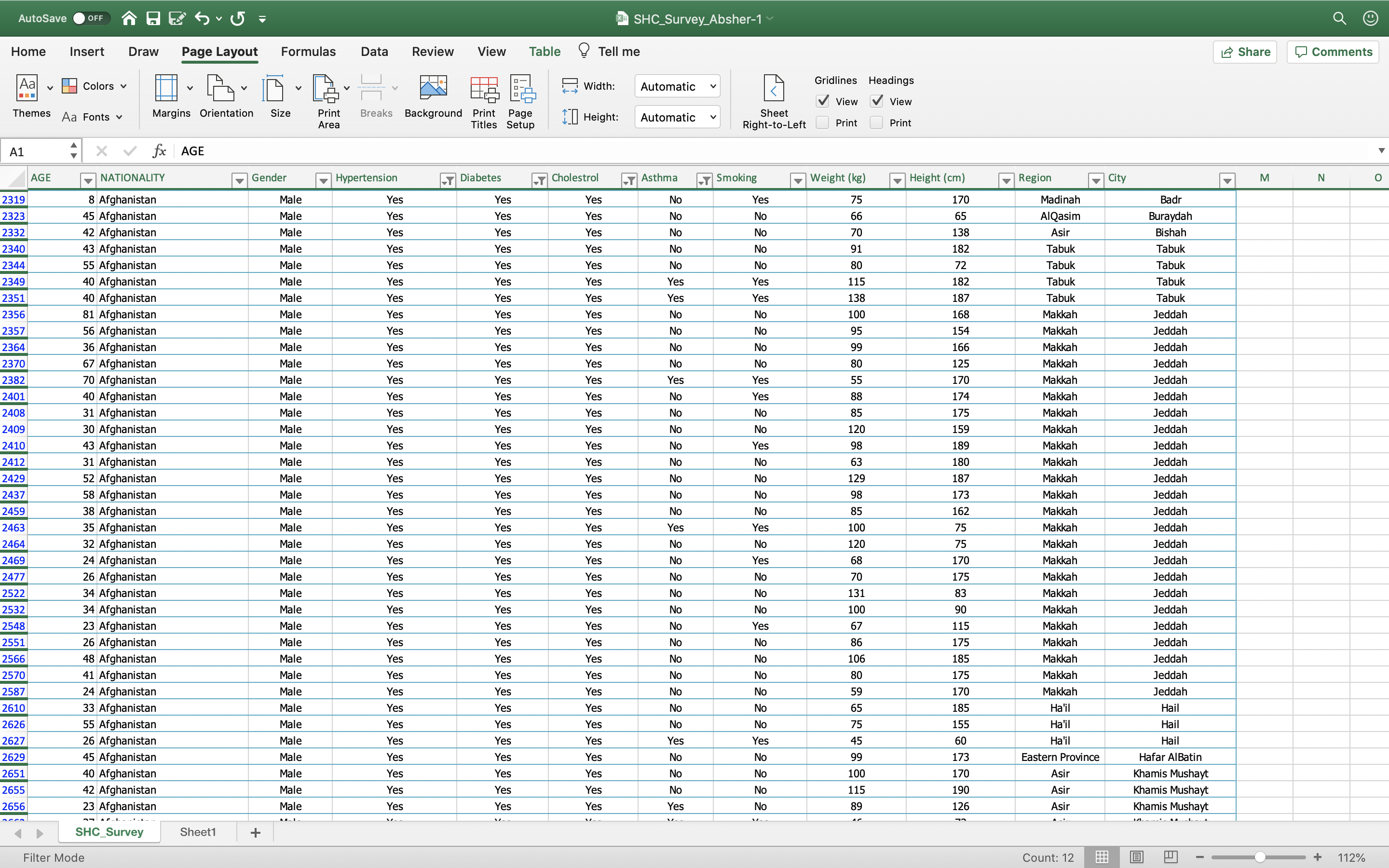

Supplement: Supplementary file 2 [file Image_1.PNG]
